# Idiosyncratic DILI: Analysis of 46,266 Cases Assessed for Causality by RUCAM and Published From 2014 to Early 2019

**DOI:** 10.3389/fphar.2019.00730

**Published:** 2019-07-23

**Authors:** Rolf Teschke

**Affiliations:** Department of Internal Medicine II, Division of Gastroenterology and Hepatology, Klinikum Hanau, Academic Teaching Hospital of the Medical Faculty, Goethe University Frankfurt, Germany

**Keywords:** drug-induced liver injury (DILI), pharmacovigilance, Roussel Uclaf Causality Assessment Method (RUCAM), idiosyncratic DILI, intrinsic DILI, liver adaption

## Abstract

One of the most difficult challenges in clinical hepatology is the diagnosis of a drug-induced liver injury (DILI). The timing of the events, exclusion of alternative causes, and taking into account the clinical context should be systematically assessed and scored in a transparent manner. RUCAM (Roussel Uclaf Causality Assessment Method) is a well-established diagnostic algorithm and scale to assess causality in patients with suspected DILI. First published in 1993 and updated in 2016, RUCAM is now the worldwide most commonly used causality assessment method (CAM) for DILI. The following manuscript highlights the recent implementation of RUCAM around the world, by reviewing the literature for publications that utilized RUCAM, and provides a review of “best practices” for the use of RUCAM in cases of suspected DILI. The worldwide appreciation of RUCAM is substantiated by the current analysis of 46,266 DILI cases, all tested for causality using RUCAM. These cases derived from 31 reports published from 2014 to early 2019. Their first authors came from 10 countries, with China on top, followed by the US, and Germany on the third rank. Importantly, all RUCAM-based DILI reports were published in high profile journals. Many other reports were published earlier from 1993 up to 2013 in support of RUCAM. Although most of the studies were of high quality, the current case analysis revealed shortcomings in few studies, not at the level of RUCAM itself but rather associated with the work of the users. To ensure in future DILI cases a better performance by the users, a list of essential elements is proposed. As an example, all suspected DILI cases should be evaluated 1) by the updated RUCAM to facilitate result comparisons, 2) according to a prospective study protocol to ensure complete data sets, 3) after exclusion of cases with herb induced liver injury (HILI) from a DILI cohort to prevent confounding variables, and 4) according to inclusion of DILI cases with RUCAM-based causality gradings of highly probable or probable, in order to increase the specificity of the results. In conclusion, RUCAM benefits from its high appreciation and performs well provided the users adhere to published recommendations to prevent confounding variability.

## Introduction

Consensus exists that patients with suspected DILI (drug-induced liver injury) require a valid diagnosis, which is emphasized also in conclusions summarized after careful analysis of the DILI case highlights published in the last years and discussed in original publications or editorials (Teschke and Andrade, 2015; Danan and Teschke, 2016; Sarges et al., 2016; Shahbaz et al., 2017; Teschke and Danan, 2017a; Real et al., 2019; Teschke, 2018a; Danan and Teschke, 2019). The first publication of the Roussel Uclaf Causality Assessment Method (RUCAM) in 1993 and its implementation in clinical routine substantially improved the causality assessment in cases of suspected DILI. This improvement and success were essentially achieved by switching from the previously used, not transparent, and vague global introspection approach to a robust, transparent, and quantitative tool providing well-defined causality gradings, which were based on the sum of individually scoring key elements. The details were recently summarized in the presentation of the updated RUCAM version (Danan and Teschke, 2016). The numerous advantages of RUCAM over alternative attempts of causality assessment methods (CAMs) explain why RUCAM is still in use for the last 25 years with continuously increased international acceptance, applicable in suspected cases of DILI and HILI (herb induced liver injury) (Danan and Teschke, 2016; Danan and Teschke, 2018; Teschke and Danan, 2018b, Teschke and Danan, 2019).

With respect to liver injury by drugs, RUCAM can help verify or dismiss causality in cases of suspected DILI and facilitates and characterizes the multiple facets of DILI. RUCAM-based DILI cases are also required to establish and validate diagnostic biomarkers (Teschke et al., 2017b), considering that the liver injury as claimed to be DILI often is not DILI but caused by diseases not related to any drug treatment (Björnsson, 2016a; Björnsson and Hoofnagle, 2016b; Teschke and Danan, 2018c; Teschke, 2018d). DILI has to be differentiated from various other liver diseases. For this differentiation, experienced physicians including hepatologists are essential, who are devoted to solve this clinical issue through individual alertness and use of a validated CAM like RUCAM in its updated version. Missing the correct DILI diagnosis may harm the patient or, in worst-case scenario, cause legal discussions.

The focus of this review is on published DILI cases, assessed for causality using RUCAM. More specifically, a list of publications containing recent 46,266 DILI cases with RUCAM-based causality assessment and a list of the top drugs implicated in DILI are presented, along with a survey of alternative causes wrongly considered as DILI. The current analysis was limited to DILI cases published within the last 5 years: from January 2014 to February 2019.

## Literature Search and Data Source

Aiming to identify publications relevant to the topic of the current review article, the database of PubMed was used for these search terms: drug-induced liver injury and DILI; both terms were used alone or in combination with RUCAM or Roussel Uclaf Causality Assessment Method. As a result, for drug-induced liver injury combined with RUCAM or Roussel Uclaf Causality Assessment Method, each provided around 33,200 hits; for DILI combined with RUCAM, around 7,070 hits were obtained, and for drug-induced liver injury together with Roussel Uclaf Causality Assessment Method, another 13,700 hits. The online search was completed on 22 February 2019.

Relevant publications of interest focusing on the topic of the article were included in the reference list. Reports in English language were preferred. The primary aim was to use original publications including case reports and case series, consensus reports, and review articles.

## Definitions

In the analysis of this large case series, definitions of terms have been used in order to ensure homogeneity of the approach.

### Liver Adaptation and Liver Injury

Provided contraindications are respected and the daily dose is within the recommended range, regulatory approved drugs are normally well-tolerated chemicals that are metabolized in the liver without harming the organ despite possible metabolic interactions. However, under certain circumstances, drugs may cause liver adaptation, also described as liver tolerance, or initiate even liver injury. Prerequisite for defining these conditions is the exclusion of alternative causes.

#### Liver Adaptation

Liver adaptation in connection with a drug therapy represents a mild modification of liver integrity, as evidenced by small increases of liver tests (LTs) that return to normal with continued drug treatment, referring to aminotransferases and/or alkaline phosphatase. Most of the commonly used drugs can presumably cause liver adaptation, although this question has rarely been extensively investigated in detail except in small samples within clinical trials. Examples for drugs causing liver adaptation include statins and isonicotinic acid hydrazine (INH; both are also known for causing also rare idiosyncratic DILI) (Björnsson, 2014; Teschke and Danan, 2016a, Teschke and Danan, 2017c; Teschke, 2018d), as well as paracetamol, which may cause liver adaptation, but mostly induces intrinsic DILI and extremely rare idiosyncratic DILI (Teschke and Zhu, 2018e). Some general features of drug-induced liver adaptation are known and listed (**Table 1**).

**Table 1:** Criteria of liver adaptation and liver injury types.

Mechanistic background	Thresholds of liver tests	Criteria and characteristic features	Recommended description
Adaptive	ALT <5 × ULN ALP <2 × ULN	• Develops at recommended daily dose • Presumably the majority of drugs have the potency of causing rare but clinically not apparent liver adaptation • Normalization or stabilization of liver tests is commonly observed whether the drug is discontinued or continued • With continuation of drug use, there is a rare risk of transition to idiosyncratic DILI	Liver adaptation
Idiosyncratic	ALT ≥5 × ULN ALP ≥2 × ULN	• Caused at recommended daily doses • Cessation of drug use is obligatory • Worsening while drug is continued • Most drugs cause rare idiosyncratic DILI, often called DILI in short if not specified • Risk of acute liver failure	Idiosyncratic DILI
Intrinsic	ALT ≥5 × ULN ALP ≥2 × ULN	• Emerges soon after acute drug overdose • Only few drugs are known for causing intrinsic DILI; antidotes may be available • Risk of acute liver failure	Intrinsic DILI

ALT, alanine aminotransferase; AST, aspartate aminotransferase; DILI, drug-induced liver injury; ULN, upper limit of normal.

#### Idiosyncratic and Intrinsic DILI

**Figure 1:**
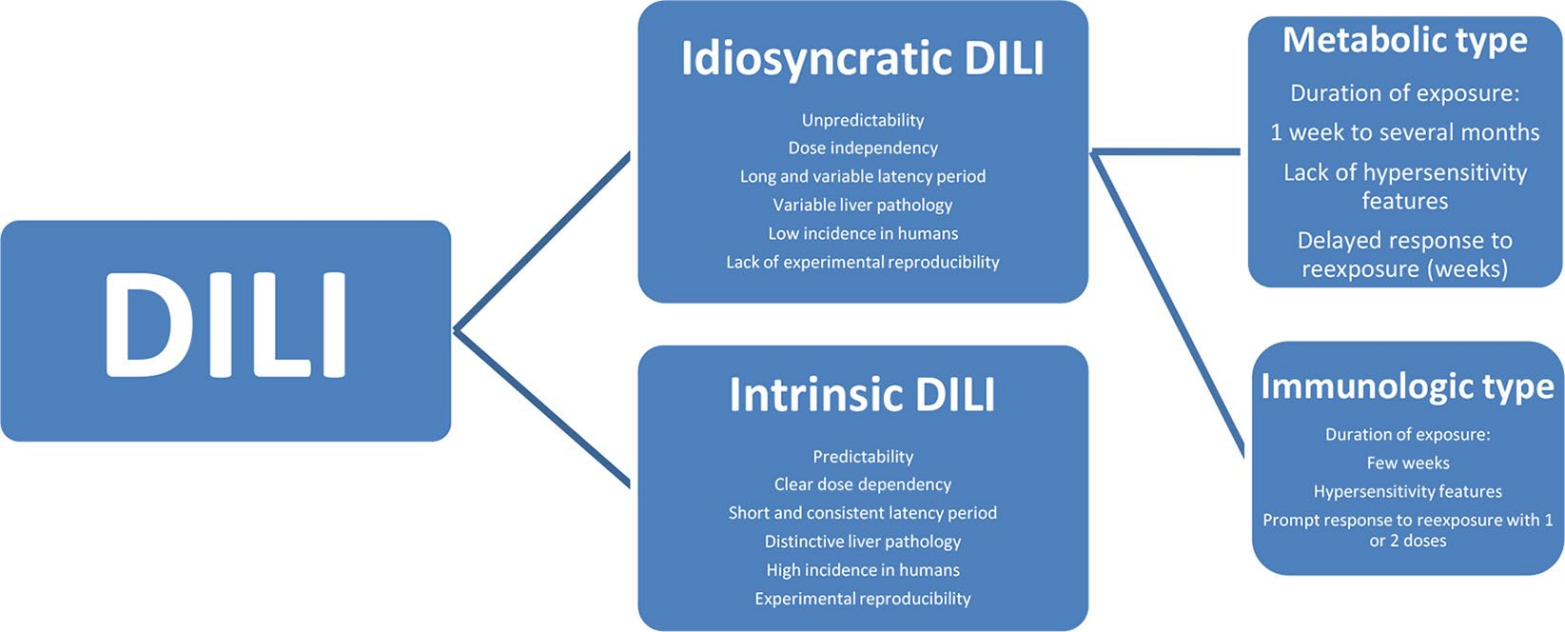
Characteristics of idiosyncratic DILI and intrinsic DILI. Reproduced from a previous report (Teschke and Danan, 2018b) with permission of the publisher Wiley-Blackwell Corporation. Abbreviation: DILI, drug-induced liver injury.

**Figure 2:**
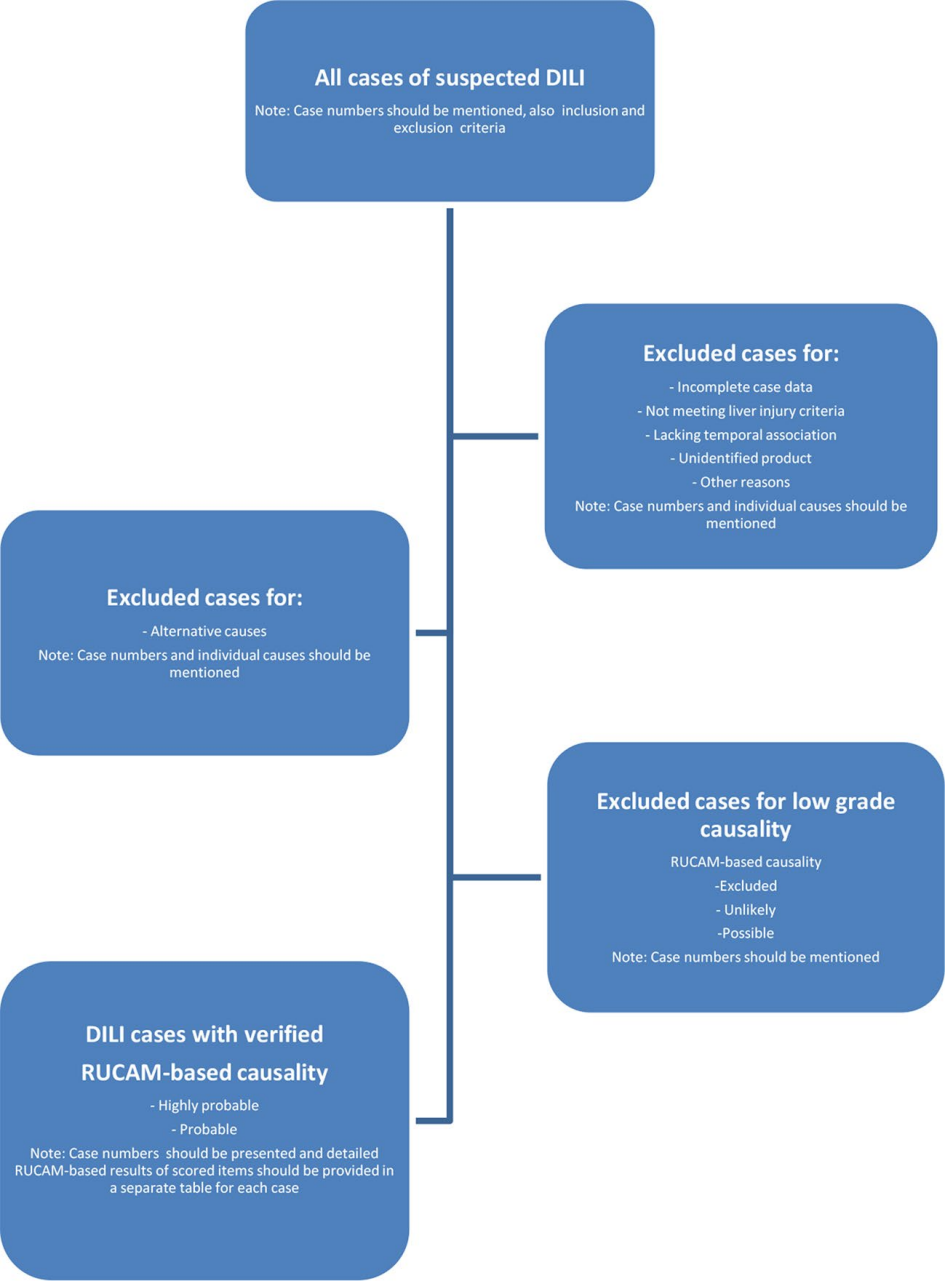
Suggestion for a diagnostic flow chart of a prospective case series DILI, in preparation of a publication. In this flow chart, DILI stand for idiosyncratic DILI. Abbreviations: DILI, drug-induced liver injury; RUCAM, Roussel Uclaf Causality Assessment Method. Modified and reproduced from a previous publication (Teschke and Danan, 2018b) with permission of the Publisher Wiley-Blackwell Corporation.

Liver injury associated with a drug treatment can be ascribed to the interaction between the drug and patient factors (idiosyncrasy) or to the drug itself (intrinsic toxicity). In practice, DILI commonly stands for the idiosyncratic DILI, the basically preferred and more specific term to avoid confusion (**Table 1**), which develops among a few individuals under the treatment with drugs used at recommended doses and is caused by unpredictable events due to immunologic or metabolic drug reactions (**Figure 1**) (Teschke and Danan, 2018b). Conditions are different for the intrinsic DILI (**Table 1**), which shows a clear dependency on the drug dose and represents therefore a predictable reaction caused by overdose of the used drugs like paracetamol, also called acetaminophen (Teschke and Zhu, 2018e). The mechanistic background differs substantially between these two DILI types (**Figure 2**) (Teschke and Danan, 2018c). In a clinical setting, the offending drug(s) often cannot be identified, problems best ascribed to vague principles of diagnosis related to undetermined DILI typology and application of CAMs, which do not follow a quantitative and transparent scoring system such as RUCAM. Problematic are reports of liver injury case cohorts in which no group differentiation was made between patients who experienced idiosyncratic DILI and those with intrinsic DILI because results obtained from these mixed patient groups are vague and disputable.

### Thresholds

Assessing the causality in suspected DILI cases begins with defining the type of liver injury (**Table 1**). Currently used criteria of a major liver injury include serum activities of LTs, namely alanine aminotransferase (ALT) of at least 5 × ULN (upper limit of normal) and/or alkaline phosphatase (ALP) of hepatic origin and at least 2 × ULN (Danan and Teschke, 2016). This ALT threshold is important to recognize early and remove all cases with minor and usually reversible liver injury from the evaluation. Diseases to be excluded are, for instance, those occurring in patients who are overweight, who are obese or morbidly obese, or who have an increased body mass index (BMI). Patients with a high BMI are at risk of developing a metabolic syndrome, nonalcoholic fatty liver disease (NAFLD), or nonalcoholic steatohepatitis (NASH). It is therefore prudent to clear these cases away from the DILI cohorts and focus on actual DILI cases, efforts that also avoid costly diagnostic procedures.

### Laboratory-Defined Liver Injury Pattern

There is also a need to determine the liver injury pattern. This can be achieved by assessing the ratio R, to be calculated through the multiple of the ULN of serum ALT divided by the multiple of the ULN of serum ALP, provided the ALP increase is of hepatic origin. The R value allows differentiation of the hepatocellular injury from the cholestatic/mixed liver injury. For each injury type, a specific RUCAM subscale is available and must be used for causality assessment. The best time assessing the ratio R is at the start of the liver injury because the initial type could evolve in the further course towards another type that would change the criteria for the causality assessment (Danan and Teschke, 2016, Danan and Teschke, 2018, Danan and Teschke, 2019). For evaluation in a normal setting, two types of liver injury are to be considered: first a hepatocellular injury with R > 5, and second, a cholestatic/mixed liver injury with R ≤ 5. This differentiation is essential because risk factors and time courses of ALT and ALP are different, and it also explains why the RUCAM scale needs two subtypes (Danan and Teschke, 2016), one for the hepatocellular injury (**Table 2**) and the other one for the cholestatic/mixed liver injury (**Table 3**).

**Table 2:** RUCAM worksheet for hepatocellular injury.

Suspected product:	Date:	
Items for hepatocellular injury	Score	Result
**1. Time to onset from the beginning of the drug/herb** • 5–90 days (rechallenge: 1–15 days) • <5 or >90 days (rechallenge: >15 days) Alternative: Time to onset from cessation of the drug/herb • ≤15 days (except for slowly metabolized chemicals: >15 days)	+2 +1 +1	□ □ □
**2. Course of ALT after cessation of the drug/herb** **Percentage difference between ALT peak and ULN** • Decrease ≥50% within 8 days • Decrease ≥50% within 30 days • No information or continued drug use • Decrease ≥50% after the 30th day • Decrease <50% after the 30th day or recurrent increase	+3 +2 0 0 -2	□ □ □ □ □
**3. Risk factors** • Alcohol use (current drinks/day: >2 for women, >3 for men) • Alcohol use (current drinks/day: ≤2 for women, ≤3 for men) • Age ≥55 years • Age <55 years	+1 0 +1 0	□ □ □ □
**4. Concomitant drug(s)/herb(s)** • None or no information • Concomitant drug/herb with incompatible time to onset • Concomitant drug/herb with time to onset 5–90 days • Concomitant drug/herb known as hepatotoxin and with time to onset 5–90 days • Concomitant drug/herb with evidence for its role in this case (positive rechallenge or validated test)	0 0 -1 -2 -3	□ □ □ □ □
**5. Search for alternative causes** **Group I (7 causes)** • HAV: Anti-HAV-IgM • HBV: HBsAg, anti-HBc-IgM, HBV-DNA • HCV: Anti-HCV, HCV-RNA • HEV: Anti-HEV-IgM, anti-HEV-IgG, HEV-RNA • Hepatobiliary sonography/Doppler/CT/MRC • Alcoholism (AST/ALT ≥2) • Acute recent hypotension history (particularly if underlying heart disease) **Group II (5 causes)** • Complications of underlying disease(s) such as sepsis, metastatic malignancy, autoimmune hepatitis, chronic hepatitis B or C, primary biliary cholangitis or sclerosing cholangitis, genetic liver diseases • Infection suggested by PCR and titer change for • CMV (anti-CMV-IgM, anti-CMV-IgG) • EBV (anti-EBV-IgM, anti-EBV-IgG) • HSV (anti-HSV-IgM, anti-HSV-IgG) • VZV (anti-VZV-IgM, anti-VZV-IgG) **Evaluation of groups I and II** • All causes—groups I and II—reasonably ruled out • The 7 causes of group I ruled out • 6 or 5 causes of group I ruled out • Less than 5 causes of group I ruled out • Alternative cause highly probable	Tick if negative □ □ □ □ □ □ □ □ □ □ □ □ □ □ □ +2 +1 0 -2 -3	Tick if not done □ □ □ □ □ □ □ □ □ □ □ □ □ □ □ □ □ □ □ □
**6. Previous hepatotoxicity of the drug/herb** • Reaction labelled in the product characteristics • Reaction published but unlabeled • Reaction unknown	+2 +1 0	□ □ □
**7. Response to unintentional re-exposure** • Doubling of ALT with the drug/herb alone, provided ALT below 5 × ULN before re-exposure • Doubling of ALT with the drug(s)/herb(s) already given at the time of first reaction • Increase of ALT but less than ULN in the same conditions as for the first administration • Other situations	+3 +1 -2 0	□ □ □ □
**Total score**		

Adapted from a previous report (Danan and Teschke, 2016). The above items specifically refer to the hepatocellular injury rather than to the cholestatic or mixed liver injury (shown in **Table 3**).

ALT, alanine aminotransferase; AST, aspartate aminotransferase; CMV, cytomegalovirus; CT, computed tomography; EBV, Epstein–Barr virus; HAV, hepatitis A virus; HBc, hepatitis B core; HBsAg, hepatitis B antigen; HBV, hepatitis B virus; HCV, hepatitis C virus; HEV, hepatitis E virus; HSV, herpes simplex virus; MRC, magnetic resonance cholangiography; ULN, upper limit of the normal range; RUCAM, Roussel Uclaf Causality Assessment Method; VZV, varicella zoster virus.

Total score and resulting causality grading: ≤0, excluded; 1–2, unlikely; 3–5, possible; 6–8, probable; ≥9, highly probable.

**Table 3:** RUCAM worksheet for cholestatic or mixed liver injury.

Suspected product:	Date:	
Items for cholestatic or mixed liver injury	Score	Result
**1. Time to onset from the beginning of the drug/herb** • 5–90 days (rechallenge: 1–90 days) • <5 or >90 days (rechallenge: >90 days) Alternative: Time to onset from cessation of the drug/herb • ≤30 days (except for slowly metabolized chemicals: >30 days)	+2 +1 +1	□ □ □
**2. Course of ALP after cessation of the drug/herb** Percentage difference between ALP peak and ULN • Decrease ≥50% within 180 days • Decrease <50% within 180 days • No information, persistence, increase, or continued drug/herb use	+2 +1 0	□ □ □
**3. Risk factors** • Alcohol use current drinks/day: >2 for women, >3 for men) • Alcohol use (current drinks/day: ≤2 for women, ≤3 for men) • Pregnancy • Age ≥55 years • Age <55 years	+1 0 +1 +1 0	□ □ □ □ □
**4. Concomitant use of drug(s)/herb(s)** • None or no information • Concomitant drug/herb with incompatible time to onset • Concomitant drug/herb with time to onset 5–90 days • Concomitant drug/herb known as hepatotoxin and with time to onset 5–90 days • Concomitant drug/herb with evidence for its role in this case (positive rechallenge or validated test)	0 0 -1 -2 -3	□ □ □ □ □
**5. Search for alternative causes** Group I (7 causes) • HAV: Anti-HAV-IgM • HBV: HBsAg, anti-HBc-IgM, HBV-DNA • HCV: Anti-HCV, HCV-RNA • HEV: Anti-HEV-IgM, anti-HEV-IgG, HEV-RNA • Hepatobiliary sonography/Doppler/CT/MRC • Alcoholism (AST/ALT ≥2) • Acute recent hypotension history (particularly if underlying heart disease) Group II (5 causes) • Complications of underlying disease(s) such as sepsis, metastatic malignancy, autoimmune hepatitis, chronic hepatitis B or C, primary biliary cholangitis or sclerosing cholangitis, genetic liver diseases • Infection suggested by PCR and titer change for • CMV (anti-CMV-IgM, anti-CMV-IgG) • EBV (anti-EBV-IgM, anti-EBV-IgG) • HSV (anti-HSV-IgM, anti-HSV-IgG) • VZV (anti-VZV-IgM, anti-VZV-IgG) Evaluation of group I and II • All causes—groups I and II—reasonably ruled out • The 7 causes of group I ruled out • 6 or 5 causes of group I ruled out • Less than 5 causes of group I ruled out • Alternative cause highly probable	Tick if negative □ □ □ □ □ □ □ □ □ □ □ □ □ □ □ □ +2 +1 0 -2 -3	Tick if not done □ □ □ □ □ □ □ □ □ □ □ □ □ □ □ □ □ □ □ □ □
**6. Previous hepatotoxicity of the drug/herb** • Reaction labelled in the product characteristics • Reaction published but unlabeled • Reaction unknown	+2 +1 0	□ □ □
**7. Response to unintentional re-exposure** • Doubling of ALP with the drug/herb alone, provided ALP below 2 x ULN before re-exposure • Doubling of ALP with the drugs(s)/herbs(s) already given at the time of first reaction • Increase of ALP but less than ULN in the same conditions as for the first administration • Other situations	+3 +1 -2 0	□ □ □ □
**Total score**		

Adapted from a previous report (Danan and Teschke, 2016). The above items specifically refer to the cholestatic or mixed liver injury rather than to the hepatocellular injury (shown in **Table 2**).

ALP, alkaline phosphatase; ALT, alanine aminotransferase; AST, aspartate aminotransferase; CMV, cytomegalovirus; CT, computed tomography; DILI, drug-induced liver injury; EBV, Epstein–Barr virus; HAV, hepatitis A virus; HBc, hepatitis B core; HBsAg, hepatitis B antigen; HBV, hepatitis B virus; HCV, hepatitis C virus; HEV, hepatitis E virus; HSV, herpes simplex virus; MRC, magnetic resonance cholangiography; ULN, upper limit of the normal range; RUCAM, Roussel Uclaf Causality Assessment Method; VZV, varicella zoster virus.

Total score and resulting causality grading: ≤0, excluded; 1–2, unlikely; 3–5, possible; 6–8, probable; ≥9, highly probable.

## RUCAM

RUCAM with its two subscales (**Tables 2** and **3**) has received overwhelming international support, as evidenced by the large number of DILI and HILI case evaluations with causality assessment by RUCAM, which were reported from 1993 until 2016 in over 100 publications (Danan and Teschke, 2016). Since 2014, RUCAM has additionally been supported by DILI experts of several countries including the US, China, and Germany. For instance, in a large US study using RUCAM in search for DILI cases in electronic medical records, 11,109 patients overall with 14,925 DILI events were found (Cheetham et al., 2014). In addition, in the US, 493 DILI cases were assessed for causality by RUCAM (Hayashi and Björnsson, 2018). It looks like that other members of the US DILIN now become more familiar with RUCAM, as helpful comments on a recent manuscript have been acknowledged in the context of the Chinese guidelines for the diagnosis and treatment of drug-induced liver injury, which recommended the use of the original RUCAM of 1993 (Yu et al., 2017) but unfortunately not the actual updated RUCAM version of 2016 (Danan and Teschke, 2016). What’s even more and substantially better, a DILIN member co-authored a large Chinese DILI paper of 18,956 DILI patients, all assessed using the original RUCAM of 1993 (Shen et al., 2019). Another member of the US DILIN group served as a senior author of a publication with focus on a critical assessment of published DILI case reports, presenting in at least 318 cases an unlikely causality grading based on the original RUCAM of 1993 (Björnsson and Hoofnagle, 2016b). Support from China came from another publication with 870 RUCAM-based DILI cases and the recommendation for general use of RUCAM in DILI case assessment (Zhu et al., 2016). Substantial support for thorough analysis of DILI cases and promotion for causality assessment by the updated RUCAM was provided by US experts of DILI from the group of James H. Lewis and his associates (Sarges et al., 2016; Shahbaz et al., 2017; Real et al., 2018).

RUCAM is appreciated as a validated, liver-specific, structured, and quantitative CAM with a clear scoring system of well-defined key elements that provide a transparent final causality grading after summing up of the individual element scores (Danan and Teschke, 2016). Compared to RUCAM at the prime position, other CAMs are poorly positioned. In particular, there is no evidence in the global DILI setting that any other CAM can presently outperform RUCAM, which represents a learning system with a biological diagnostic background that represents a limitation of RUCAM use. Out of these reasons, RUCAM is not necessarily perfect in covering all tentative diagnostic aspects related to the variability of DILI features and covering all of the more than 1,000 different drugs implicated in causing liver injury (Teschke and Danan, 2018b). Certainly, a variety of other CAMs are on the market with their major shortcomings, which have been outlined recently (Danan and Teschke, 2016; Teschke and Danan, 2018b). In brief, some CAMs are not specific for the liver and liver injury; others are poor plagiates of the original RUCAM, confounded by deleting and modifying its original elements or adding new elements with not validated criteria and scoring. Other CAMs claimed having incorporated RUCAM elements, but by checking the conditions, it turned out that element criteria were insufficiently transferred from the RUCAM system, or the RUCAM specific scoring system was evidently omitted. Despite these deletions, new global causality gradings were offered as percentage ranges that may erroneously be misinterpreted as the result of obtained individual element scoring, which in fact was not done. Finally, CAMs based on global introspection are an additional problem because assessment is subjective and not transparent, not based on valid element criteria, and devoid of a validated scoring system. In essence, only RUCAM is seemingly in the comfortable situation to assist establishing causality in assumed DILI cases. No other CAM has a similar successful clinical and scientific run, and the lack of such a background and accuracy prevents any attempt of overriding and outperforming the successful RUCAM.

In line with other CAMs, RUCAM shares the problem of DILI diagnosis in patients with hepatitis B or C, which requires assistance by an experienced virologist to assess the viral infection and importance of the viral load as contributory or sole factor of the disease. In general, cases with preexisting liver disease require special clinical attention because for these an individual RUCAM scoring system is not available, but general recommendations have been published (Danan and Teschke, 2016). Previously, the issue of diagnosis and management of acute idiosyncratic DILI in patients with preexisting liver disease has thoroughly been analyzed and discussed (Teschke and Danan, 2016a). Regarding clinical trials using patients with preexisting liver disease, a pragmatic approach suggests subtracting baseline reference LT values before start of the trial from actual LT values obtained during the trial, because this would bring evidence whether the thresholds of ALT >5 × ULN or ALP >2 × ULN are fulfilled for the suspected DILI (Teschke and Danan, 2016a), and the available data can then undergo causality assessment based on the updated RUCAM (Danan and Teschke, 2016; Teschke and Danan, 2016a). Of note, RUCAM has no problem assessing drugs with unknown previous liver events (drugs in clinical trials) or newly marketed drugs like immune modulating agents and more specifically immune checkpoint inhibitors because it considers these drug-related limitations appropriately by not providing high causality gradings due to few missing elements and their individual scores.

## Global DILI Cases Assessed by RUCAM

### Large Case Series

The present analysis of selected 36 reports provided by authors originating from 10 countries published from 2014 until end of February 2019 revealed that 46,266 DILI cases had been evaluated for causality by one of the two latest RUCAM versions of 1993 and 2016 (**Table 4**). This allows a good overview on the quality and shortcomings of DILI case assessment using RUCAM and merits some comments on the published reports (**Table 4**):

1. The current listing of RUCAM-based DILI cases is large (**Table 4**) and confirms previous impressions that RUCAM is well accepted in the scientific community of DILI experts (Danan and Teschke, 2016). The updated RUCAM of 2016 has increasingly been used in the last few years (**Table 4**) and should be the preferred RUCAM version in future DILI case assessments.
2. With few exceptions, drug groups or individual drugs were specified; this applies preferentially to cohorts of small or intermediate size, which often present detailed information on characteristic features of DILI caused by specific drugs, while large cohorts commonly failed to differentiate between individual drugs but provide a broader overview.
3. In most reports, final RUCAM scores with corresponding RUCAM-based causality gradings were carefully listed, allowing thereby some conclusions on case data quality. In general, high numbers of DILI cases with a possible causality grading commonly reflect incomplete case data sets because RUCAM includes this condition by providing low scores for missing key elements.
4. High causality gradings of probable or highly probable likelihood are commonly achieved with prospective studies that facilitate collection of complete data in time while the patient is under medical care. Consequently, prospective studies are preferred over retrospective ones because their results may be a matter of discussion.
5. Most reports present clear and precise data on DILI related to synthetic chemical drugs, allowing valid description of DILI features and discussion about other points of interest like aspects of epidemiology. Such data may differ from country to country, but conclusions should be valid as all studies were based only on RUCAM.
6. Publications with cohorts consisting of both DILI and HILI cases create confusion unless groups are separately considered prior to final analysis.
7. In the majority of reports, the RUCAM version used was specified with preference of the original RUCAM of 1993 and the now preferred updated RUCAM of 2016, whereas initial versions of 1990 or before are clearly incomplete.
8. Occasionally, reports came along without specifying the RUCAM version used or even without referencing the respective publication, to be viewed as a major deficiency. Some authors even misquote or fail to reference the correct original or updated RUCAM version or quote instead a secondary literature source that has nothing to do with the original one. Although DILI researchers and clinicians are likely familiar with RUCAM and its correct reference source, others may not and would appreciate a correct quotation.

**Table 4:** List of selected publications and analyses from national registries and medical centers that applied RUCAM in suspected DILI cases included in major case series and published from 2014 to early 2019.

First author	Country	Center or Registry	Drugs	DILI Cases (n)	Details and comments
Bohm et al., 2014	USA	South Carolina College of Pharmacy	Daptomycin	9	Causality assessment by RUCAM but without specified score and by other CAMs.
Cheetham et al., 2014	USA	Kaiser Permanente Southern California, Pharmacy Analytical Services	Multiple drugs	11,109	Using an electronic version of RUCAM, overall 11,109 patients with 14,925 DILI events were assessed, categorized for 15.5% as probable or highly probable, 59.6% as possible, and 24.9% as unlikely to the drug.
Douros et al., 2014	Germany	Berlin Case-control Surveillance Study	Multiple drugs	198	Retrospective analysis of 198 DILI cases after exclusion of alternative causes. RUCAM-based causality showed causality grading of highly probable, probable, and possible.
Russmann et al., 2014	Switzerland	University Hospital Zurich	Rivaroxaban	14	Based on the causality assessment by the original RUCAM of 1993, causality grading was highly probable in 4 patients, probable in 7 patients, and possible in 3 patients.
Russo et al., 2014	USA	Carolinas Medical Center, Charlotte, NC	Various statins	22	With the original RUCAM of 1993, causality gradings were highly probable in 4 patients, probable in 16 patients, and possible in 2 patients.
Ou et al., 2015	China	Central South University, Changsha	Multiple drugs	231	Used RUCAM-based criteria and assessed causality by RUCAM, but no reference is provided. In addition, 130 HILI cases by herbal TCM were published also assessed by RUCAM.
Robles-Diaz et al., 2015	Spain and Latin America	Spanish DILI Registry and Spanish-Latin-American DILI Network	Anabolic and androgenic steroids	25	Analysis of cases retrieved from 2 prospective databases. Only cases were presented as being drug-related based on causality assessment by RUCAM, but specific causality gradings were not presented for individual products although specifically itemized.
Zhu et al., 2015	China	302 Military Hospital Beijing	Various drugs	39	DILI study in children, using for causality assessment the original RUCAM of 1993.
Bessone et al., 2016	Argentina	University of Rosario School of Medicine	Various drugs	197	Based on the original RUCAM of 1993, causality was highly probable (9%), probable (67%), and possible (24%).
Lu et al., 2016	China	Third People´s Hospital of Changzhou, Changzhou	Various drugs	172	Used all DILI criteria of the original RUCAM of 1993, viewed as a universally recognized method for evaluating DILI. Separately, 252 cases of HILI by herbal TCM had been evaluated.
Medina-Caliz et al., 2016	Spain	Hospital Virgen de la Victoria, University of Malaga	Various drugs	298	Use of the original RUCAM of 1993: highly probable causality in 43% of the patients, probable in 50%, and possible in 7%.
Yang et al., 2016	China	Shengjing Hospital, China Medical University, Shenyang	Various drugs	124	Retrospective study with inclusion of all DILI cases with a RUCAM score ≥6 based on the original RUCAM of 1993.
Zhu et al., 2016	China	Specialized Committee for Drug-induced Liver Injury, Chinese Pharmacological Society, Beijing	Multiple drugs	870	Retrospective study of hospitalized patients with DILI by Western drugs and careful exclusion of alternative causes. Most drugs were specified in a listing. For all DILI cases, causality assessment using RUCAM provided causality gradings mostly of highly probable or probable and rarely of possible.
Abbara et al., 2017	United Kingdom	Northwick Park Hospital	Anti-tuberculotics	105	Single center retrospective study using the updated RUCAM version: All patients received at least a possible scoring, more than half a probable one.
Ferrajolo et al., 2017	Italy	University of Campania, Naples	Multiple antibiotics	938	Pediatric cases. Use of the CIOMS/RUCAM version of 1990, which is somewhat outdated now in face of the original RUCAM of 1993 or the updated RUCAM of 2016.
Licata et al., 2017	Italy	University of Palermo		185	RUCAM discussed and used for causality assessment.
Naiqiong et al., 2017	China	Fuwai Hospital Chinese Academy of Medical Sciences, Beijing	Statins	157	Cases with a probable or highly probable causality grading, assessed using the original RUCAM of 1993. Randomized controlled trial of Bycyclol for treating DILI.
Nicoletti et al., 2017	USA and other countries	Department of Systems Biology, Columbia University, New York, NY	Multiple drugs	339	Partially retrospective study, using unlear version of RUCAM with inclusion of RUCAM based causality gradings of possible and higher. Data confounded by additional application of global introspection.
Rathi et al., 2017	India	Lokmanya Tilak Municipal Medical College, Sion, Mumbai, Maharashtra	Multiple drugs	82	Prospective study considering DILI cases with RUCAM-based causality gradings mostly of highly probable or probable, and rarely of possible. Initially, alternative causes had carefully been excluded. Perfect study.
Das et al., 2018	India	Jawaharlal Institute of Postgraduate Medical Education, Puducherry	Several drugs	24	Causality assessment by the original RUCAM of 1993 and the updated RUCAM of 2016, providing all causality gradings but in 14 cases a probable grading.
Dragoi et al., 2018	Germany	University Hospital Munich	Diclofenac	16	Reported causality assessment included the use of the updated RUCAM, and all cases were classified as having for DILI by diclofenac a likelihood of at least “highly likely” that is not a causality grading of RUCAM. Uncertainty remains also regarding the expression of “at least” that would imply tentative higher causality gradings that in fact do not exist. Applied re-exposure criteria remained unreported.
Giacomelli et al., 2018	Italy	DIBIC Luigi Sacco, University of Milan	Nevirapine	8	Causality assessment using the updated RUCAM revealed a possible causality in 3 patients and a probable one in 5 patients.
Hayashi and Björnsson, 2018	USA and Europe	University of North Carolina, Chapel Hill, and National University Hospital of Iceland, Reykjavik	Unspecified	493	Reported as highly probable and probable causality according to not referenced and not specified version of RUCAM.
Tao et al., 2018	China	Nanjing Medical University, Nanjing	Anti-tuberculotics	290	Use of the updated RUCAM of 2016: 174 patients received a possible causality grading, 116 patients a probable one.
Teschke, 2018a	Germany	Klinikum Hanau, Goethe University Frankfurt/Main	Multiple drugs	7,278	All these cases received a RUCAM-based causality assessment, derived from a cohort of overall 13,335 patients from which 6,057 patients have been deducted because for these causality evaluations were not based on RUCAM.
Teschke and Danan, 2018b	Germany	Klinikum Hanau, Goethe University Frankfurt/Main	Multiple drugs	3,312	RUCAM-based DILI cases as published by registries and major clinical centers, currently used to establish a list of the 10 drugs most commonly incriminated in DILI.
Wang et al., 2018	China	Zhejing University, Hangzhou	Anti-tuberculotics	155	RUCAM cores were highly probable in 22.85% of the patients, probable in 56.77%, and possible in 20.65% of the patients, but the RUCAM version used was not clearly identified in the text.
Aiso et al., 2019	Japan	Teikyo University School of Medicine, Tokyo	Multiple drugs	270	Prospective study using the original RUCAM of 1993 provided causality gradings of highly probable in 49% of the patients, probable in 40%, possible in 11%, and unlikely in 1% of the patients.
Bessone et al., 2019	Argentina	University of Rosario, School of Medicine	Multiple drugs	114	Initially 311 cases, from which 197 cases had to be subtracted as published already in 2016. RUCAM considered as the best scoring system for DILI was used but version not specified and referenced.
Kwon et al., 2019	Japan	University of Tsukuba	Nimesulide	33	Based on the updated RUCAM of 2016, causality was highly probable in 11 patients, probable in 18 patients, and possible in 4 patients. Perfect study, allowing thorough description of DILI by Nimesulide.
Nicoletti et al., 2019	USA and other countries	Department of Systems Biology, Columbia University, New York, NY	Flucloxacillin	197	Likely at least partially retrospective study using possibly the original RUCAM version of 1993 providing causality gradings of possible or higher. Results confounded by additional use of global introspection.
Shen et al., 2019	China	Overall 308 participating centers from China	Multiple drugs	18,956	Retrospective study of RUCAM-based DILI cases with causality gradings of probable and highly probable in 52% and of a possible grading in as much as 48%, without known causes for this high percentage. Considered were the years from 2012 to 2014. The initial study cohort of 25,927 included also HILI cases (26.81%), leaving 18,956 real DILI cases.
**Total cases:** **46,266**					

The CIOMS/RUCAM version of 1990 refers to Bénichou (1990); the original RUCAM of 1993 refers to the publication of Danan and Bénichou (1993); the updated RUCAM version was published by Danan and Teschke (2016).

DILI, drug-induced liver injury; RUCAM; Roussel Uclaf Causality Assessment Method.

No question, many RUCAM reports of DILI are of high quality, especially those of national DILI registries in Europe, which assessed cases prospectively and provided excellent results with high causality gradings and details as referenced previously (Danan and Teschke, 2016; Teschke, 2018d). In addition, authors from India published another prospective study on DILI with high causality gradings (Rathi et al., 2017), classified as a report of excellence (Teschke and Danan, 2017a). Applying RUCAM prospectively in the Indian cohort study facilitated early detection of non-drug causes in eight patients. Among the alternative diagnoses were acute hepatitis E virus (HEV) in three patients, autoimmune hepatitis in two patients, and hepatitis A, B, and sarcoidosis in one patient each (Rathi et al., 2017). In all patients of the Indian study, infections by HEV were systematically excluded, not only because HEV is endemic in India but also because consensus exists among experts in the field that HEV infections must be excluded in any suspected DILI or HILI case. This study from India confirmed that alternative causes can be excluded only if the patients are correctly investigated at the early phase of the liver injury.

The analysis of the 10 countries, in which the authors of the 31 reports had their working place, showed China on top, followed by the US and Germany (**Table 5**). In some reports, authors came from different countries but this is not separately listed in the table. A listing with authors of RUCAM-based DILI and HILI cases from many more countries has previously been provided (Danan and Teschke, 2016). Importantly, RUCAM-based DILI reports have been published in journals considered as one of the most relevant ones (**Table 5**), suggesting that their editors perhaps appreciate RUCAM or are at least familiar with it.

**Table 5:** Ranking of countries from which first authors reported DILI cases assessed by RUCAM as published from 2014 to early 2019 with specification of the publishing journal.

Ranking	Country	Total DILI Cases (n)	Individual DILI Cases (n)	Reporting first author
1.	China	20,994	231 39 172 124 870 157 290 155 18,956	Ou et al., 2015 Zhu et al., 2015 Lu et al., 2016 Yang et al., 2016 Zhu et al., 2016 Naiqiong et al., 2017 Tao et al., 2018 Wang et al., 2018 Shen et al., 2019
2.	USA	11,633	9 11,109 22 493	Bohm et al., 2014 Cheetham et al., 2014 Russo et al., 2014 Hayashi and Björnsson, 2018
3.	Germany	10,804	198 16 7,278 3,312	Douros et al., 2014 Dragoi et al., 2018 Teschke, 2018a Teschke and Danan, 2018b
4.	Italy	1,131	938 185 8	Ferrajolo et al., 2017 Licata et al., 2017 Giacomelli et al., 2018
5.	Spain	323	25 298	Robles-Diaz et al., 2015 Medina-Caliz et al., 2016
6.	Japan	303	270 33	Aiso et al., 2019 Kwon et al., 2019
7.	Argentina	311	197 114	Bessone et al., 2016 Bessone et al., 2019
8.	India	106	82 24	Rathi et al., 2017 Das et al., 2018
9.	United Kingdom	105	105	Abbara et al., 2017
10.	Switzerland	14	14	Russmann et al., 2014

### Single Case Reports and Small Case Series

As compared to large DILI case series, the clinical value of RUCAM-based DILI cases published as single case report or as small case series should not be underestimated; some of these had been selected as examples (**Table 6**). They usually provide a broad narrative of the DILI cases and often include for the cases a separate list with RUCAM elements and the respective scores. Using this approach, the case description itself benefits from transparency that cannot be provided for DILI cases from large case series. Therefore, short case series or single case reports are of particular value for physicians in search for details. Such informative reports of DILI cases with affirmed diagnosis should have a chance being included for instance in the LiverTox database, provided the case had been evaluated by RUCAM and qualified for a high causality grading. The impression prevails that within the last few years, the updated RUCAM is more often used (**Table 6**), good news not only for the updated RUCAM but also for the scientific community of individuals involved in DILI case assessment and management.

**Table 6:** List of offending drugs in selected case reports or small case series that applied RUCAM in suspected DILI cases, published from 2014 to early 2019.

First author	Country Region	Center or Hospital	Drugs	DILI Cases (n)	Details and comments
Veluswamy et al., 2014	USA	Icahn School of Medicine at Mount Sinai, New York, NY	Pomalidomide	1	Using RUCAM, non-drug causes were ruled out but causality grading for Pomalidomide was not reported.
Moreno et al., 2015	Spain	Corporació Sanitària Parc Taulí, Sabatell, Barcelona	Ciprofloxacin (initial), later amoxicillin + clavulanic acid	1	Case received a highly probable causality grading for ciprofloxacin, based on a RUCAM scoring of 9. However, the reference of the used RUCAM version is missing. A subsequent treatment with amoxicillin + clavulanic acid resulted in another DILI with a highly probable causality grading, again assessed by RUCAM.
Tang et al., 2015	USA	National Institutes of Health, Bethesda, MD	Bupropion doxycycline	1	Using the original RUCAM version of 1993, a probable causality grading is provided for each drug.
Alhaddad et al., 2017	Egypt	Menouffia University	Amoxicillin/clavunalate	1	RUCAM-based probable causality with a RUCAM score of 8.
Baig et al., 2015	USA	UAB Health Center Montgomery	Rivaroxaban	1	RUCAM-based score was 6, corresponding to a probable causality.
Forner et al., 2017	Canada	Dalhousie University Halifax	Ramipril	1	Probable causality, based on a RUCAM score of 7.
Niijima et al., 2017	Japan	Kan-etsu Chuo Hospital	Ipragliflozin	1	Probable causality based on a RUCAM score of 7, assessed with the updated RUCAM of 2016.
Taneja et al., 2017	India	Postgraduate Institute of Medical Education and Research, Chandigarh	Etodolac	2	Probable causality with RUCAM scores of 7 and 8, using the updated RUCAM that was correctly quoted.
Terziroli Beretta-Piccoli et al., 2017	Switzerland	Epatocentro Ticino, Lugano	Atovaquone/Proguanil	1	Assessment using the updated RUCAM, which provided a score of 10 corresponding to a highly probable causality.
Lammel-Lindemann et al., 2018	Mexico	Hospital Zambrano Hellion, San Pedro Garza García	Candesartan	1	Assessed using the updated RUCAM of 2016, which provided a RUCAM score of 9 and thereby highly probable causality grading.
Li et al., 2018	China	First Affiliated Hospital of Anhui Medical University, Hefei	Iguratimod	1	Using the updated RUCAM of 2016, causality was highly probable based on a RUCAM score of 9.
Patel et al., 2018	USA	University of California, San Diego, CA	Everolimus	1	Rather than using the updated RUCAM of 2016, the case was assessed for causality using the RUCAM of 1993, which provided a probable causality grading based on a RUCAM score of 8.
Liao et al., 2019	Taiwan	Taipei Tzu Chi Hospital, New Taipei	Cefepime	1	Using the updated RUCAM of 2016, a probable causality was achieved based on a RUCAM score of 7.
**Total cases:** **15**					

DILI, drug-induced liver injury; RUCAM, Roussel Uclaf Causality Assessment Method.

### Comments and Encouragements

The worldwide success of RUCAM is overwhelming, as evidenced by the large number of DILI cases assessed by RUCAM (**Table 4**). Analyzing the listed reports in detail, it seems that the use of RUCAM goes smoothly, more or less without major difficulties. Nevertheless, a few reports show shortcomings in case presentations and provide critical comments, which merit attention and are presented in condensed form, drawn from selected publications to provide an unbiased overview (**Table 7**). Included in the list are also encouraging statements that are refreshing. Overall, the impression prevails that RUCAM including its updated version is on the winning avenue.

**Table 7:** Comments and encouragements based on reports published from 2014 until early 2019.

First author	Country	Center or Hospital	Details and comments
Chalasani et al., 2014	USA	Indiana University School of Medicine, Indianapolis, IN	**Ambiguity:** The original RUCAM of 1993 is quoted and discussed but not specifically recommended for its use. However, published are RUCAM scales, details of RUCAM-based liver injury classification R value.
Chen et al., 2014	USA	US Food and Drug Administration, Jefferson, AR	**Issue of biomarkers with RUCAM:** Referenced were several reports dealing with RUCAM, but a practical approach is lacking how biomarkers can validly be established using cases with firm DILI.
Lim et al., 2014	USA	John Hopkins University School of Medicine, Baltimore, MD	**Confounding variables:** RUCAM was referenced and used, but additional CAMs confounded the results.
Hernández et al., 2014	Uruguay	University of Montevideo	**Weak recommendation:** RUCAM was used in rare cases from Latin American countries; referenced is the original RUCAM of 1993 that is recommended as it may improve the consistency of judgments and help obtain a more accurate DILI diagnosis.
Hornby et al., 2014	United Kingdom	University of Liverpool	**Inconsistency:** Original RUCAM of 1993 was referenced and discussed, but uncertainty how to validate microRNA as diagnostic DILI biomarker.
Regev et al., 2014	USA	Eli Lilly Pharmaceutical Company, Indianapolis, IN	**Misconception:** Original RUCAM of 1993 and RUCAM-based DILI criteria were referenced, but erroneously assumed as inferior as compared to global introspection that is negatively viewed by others as largely subjective and suffering from interobserver variability.
Senior, 2014	USA	Food and Drug Administration, Silver Spring, MD	**Encouragement:** RUCAM is viewed and referenced as a CAM for DILI used widely in Europe and elsewhere in the world.
Seyfarth et al., 2014	Germany	University Hospital of Leipzig, Leipzig	**Uncertainty:** RUCAM is viewed as the usual tool to assess causality in DILI but was not formally applied in suspected DILI by Bosental; causality was rather assumed.
Chalasani et al., 2015	USA	Indiana University School of Medicine, Indianapolis, IN	**Inconsistency:** RUCAM-based criteria of liver injury pattern and RUCAM-based R value were used, correctly quoting the original RUCAM of 1993 but its criteria for causality were not applied.
Chang et al., 2015	Taiwan	National Taiwan University, Taiwan	**Misfortune:** A large study on liver injury by statins, in which causality assessment by RUCAM was not possible that would have strengthen the impact of the results of this population-based cohort.
Hayashi et al., 2015	USA	University of North Carolina, Chapel Hill, NC	**Encouragement:** RUCAM is viewed as the most widely accepted and validated instrument for causality assessment of DILI cases, not presenting any evidence based superiority of other CAMs over RUCAM.
Khoury et al., 2015	Israel	Hadassah Medical Center, Jerusalem	**Ambiguity:** RUCAM-based thresholds, liver injury pattern, causality grading, and R value were quoted, referencing the RUCAM of 1993, but not applied.
Moreno et al., 2015	Spain	Corporació Sanitària Parc Taulí, Sabatell, Barcelona	**Insufficiency:** RUCAM was used but no reference was provided. In fact, most DILI experts know RUCAM and the special journal(s) where it is published, but some may not.
Watkins, 2015	USA	University of North Carolina, Chapel Hill, NC	**Uncertainty:** Referencing RUCAM of 1993; regrets have been expressed that in this standard diagnostic RUCAM instrument no DILI signatures had been incorporated in addition to the available hepatocellular, cholestatic, or mixed typology of liver injury. However, no suggestions were made how to define DILI signatures and how to score these.
Weiler et al., 2015	Switzerland	University Hospital Zurich, Zurich	**Ambiguity:** RUCAM-based liver injury types and R value were mentioned with original RUCAM of 1993 and quoted but not suggested for assessing diagnostic biomarkers.
Andrade et al., 2016	Spain	Hospital Virgen de la Victoria, University of Malaga	**Uncertainty:** For prospective DILI consortia, among others, also RUCAM as the standard CAM will be used, but a correct reference for the respective RUCAM version was not provided.
Benesic et al., 2016	Germany	University Hospital Munich	**Ambiguity:** Although RUCAM is viewed as the most widely used current causality assessment tool, expert opinion was classified as gold standard. For own cases, use of an unclear and mixed classification system to diagnose DILI.
Björnsson, 2016a	Iceland	National University of Iceland, Reykjavik	**Conflicting data:** Among published 671 DILI cases, a few cases received a possible causality grading using the updated RUCAM of 2016, which was not applied in the majority of the DILI cases.
Björnsson and Hoofnagle, 2016b	Iceland and USA	National University of Iceland Reykjavik and National Institutes of Diabetes and Kidney Disease, NIH, Bethesda	**Disputable recommendations:** LiverTox database contained 671 eligible DILI cases, for which the original RUCAM of 1993 was applied to a few cases to assess causality but not to the majority of cases; instead, causality for most drugs was assumed depending on the number of reported cases, meaning high numbers equal established causality that represents a highly disputable quantitative but by no means an acceptable approach.
Kim and Naisbitt, 2016a	Korea	Ajou University School of Medicine, Suwan	**Encouragement:** Original RUCAM of 1993 is referenced and RUCAM-based DILI criteria such as liver injury types and R value are mentioned and suggested to be used in genetic studies.
Kim et al., 2016b	Korea	Gyeonsang National University Hospital, Gyeonsang	**Shortcoming:** RUCAM-based criteria of DILI are used but original RUCAM of 1993 is not quoted.
Matanovic, 2016	Croatia	University Hospital Osijek	**Encouragement:** RUCAM is viewed as the best method to assess DILI cases.
Morales et al., 2016	Colombia	University of Antioquia, Medellin	**Promotion:** Of the several scales developed to assess the likelihood of DILI, RUCAM is viewed as the most widely used and best validated one.
Ortega-Alonso et al., 2016	Spain	Hospital Virgen de la Victoria, University of Malaga	**Forgotten quotation:** Derived from the original RUCAM version, several items like R value and liver injury types were mentioned without referencing the original report of 1993.
Robles-Diaz et al., 2016	Spain	Hospital Virgen de la Victoria, University of Malaga	**Insufficiency:** RUCAM-based DILI criteria such a liver injury pattern and R value were used, referring to RUCAM of 1990, but for case analysis and causality assessment RUCAM was not applied, lack of causality gradings.
Sobhonslidsuk et al., 2016	Thailand	Mahidol University, Bangkok	**Misfortune:** The updated RUCAM was viewed as a well-established, structured and standardized tool to assess causality in DILI, but was not used in the population-based study for unknown reasons.
Wang et al., 2016	Taiwan	Wei Gong Memorial Hospital, Miaoli, Taiwan	**Ambiguity:** RUCAM version of 1990 and its criteria of DILI were discussed but RUCAM itself was not used for causality assessment in this published study that otherwise did not benefit from any other CAM.
Alempijevic et al., 2017	Serbia	University of Belgrade, School of Medicine	**Fulfilled:** The original RUCAM version of 1993 was referenced also regarding its widespread use today, with a reminder of reevaluation and revision, considered as overdue. In fact, an updated RUCAM version has been available online already in 2016 (Danan and Teschke, 2016).
Amagon et al., 2017	Nigeria	College of Medicine, University of Lagos	**Ineffectivity:** Described were 285 DILI cases, not assessed by any CAM including RUCAM, which is recommended to properly establish cause and effectively quoting papers that discussed usage details of RUCAM (Teschke et al., 2016b, Teschke and Danan, 2017c).
Andrade, 2017	Spain	Hospital Virgen de la Victoria, University of Malaga	**Incomplete:** Published was that the database automatically calculates a RUCAM score for each DILI case, but the RUCAM version to be used was not quoted.
Iorga et al., 2017	USA	University of Southern California, Los Angeles, CA	**Discussion:** Updated RUCAM of 2016 and other CAMs discussed in relation to cascade of DILI events and diagnostic biomarkers but not used.
Kim et al., 2017	Korea	Catholic University of Korea, Gyeonggi-do	**Promotion:** RUCAM-based DILI types are discussed and quoted.
Kullak-Ublick et al., 2017	Switzerland	University Hospital Zurich, Zurich	**Uncertainty:** The updated RUCAM of 2016 is quoted and discussed, also in comparison to expert opinion that carries the risk of overruling a more insightful minority opinion. Advice is given to evaluate new biomarkers in patients with RUCAM-based DILI. No convincing evidence is presented that tests with MH cells do outperform the RUCAM scale, as claimed.
Licata et al., 2017	Italy	University of Palermo, Palermo	**Vague:** RUCAM of 1993 is referenced and its DILI pattern is used, but not its causality grading. Data remain vague.
Marrone et al., 2017	Italy	Catholic University of the Sacred Heart, Rome	**Promotion:** Discussed were various RUCAM-based DILI criteria, whereby RUCAM is viewed as probably the most accurate and reproducible CAM for DILI, and is among the most used and widely accepted tools.
McEuen et al., 2017	USA	US Food and Drug Administration, Jefferson, AR	**Partial success:** RUCAM was obviously applied in only some DILI cases, but for future cases the updated RUCAM version was recommended to verify causality.
Raschi and De Ponti, 2017	Italy	Department of Medical and Surgical Sciences, University of Bologna, Bologna	**Promotion:** With respect to RUCAM and quoting the updated RUCAM of 2016, it was outlined that according to major experts in the field, RUCAM has the potential of a standard scale for DILI and HILI causality assessment.
Wang et al., 2017	China	Rizhao People’s Hospital, Rizhao	**Partial success:** RUCAM-based criteria of DILI were used but source was not quoted, and no causality assessment by RUCAM or any other CAM was performed in 395 suspected DILI cases. All cases had been classified as DILI and enrolled “if they had a strong history that the liver injury was caused by a medication or an herbal medicine within 6 months before admission.”
Yamashita et al., 2017	Japan	Graduate School of Medical Sciences, Kumamoto	**Progress:** Short review on DILI, with discussion on and mentioning of RUCAM-based criteria according to the original RUCAM of 1993.
Yu et al., 2017	China	Nanjing University of Chinese Medicine, Nanjing	**Excellence:** CSH guidelines for the diagnosis and treatment of drug-induced liver injury recommend the use of RUCAM for DILI causality assessment, referencing also the updated RUCAM of 2016.
Hunt, 2018	USA	Duke University Medical Center, Durham, NC	**Encouragement:** Editorial discusses RUCAM details and recommends the use of the updated RUCAM for causality assessment in DILI cases to avoid DILI misdiagnosis.
Liu et al., 2018	China	Tianjin Medical University, Tinjian	**Progress:** RUCAM is carefully discussed in the context of biomarkers including miRNA, referencing also miRNA in cases of intrinsic DILI by paracetamol that received the highest RUCAM causality grading.
Meunier and Larrey, 2018	France	Montpellier School of Medicine, Montpellier	**Fair:** RUCAM was applied in many published cases of DILI caused by nonsteroidal anti-inflammatory drugs; the updated RUCAM of 2016 was referenced.
Patel et al., 2018	USA	University of California, San Diego, CA	**Unclarity:** RUCAM was criticized by saying it would not account for differences in type or quantity of publications, but suggestions for improvement regarding specific criteria and item scoring based on clear evidence were not provided.
Schueller et al., 2018	Germany	University Hospital RWTH Aachen	**Excellence:** RUCAM-based DILI diagnosis may soon be supported by miRNA use.
Tan et al., 2018	Singapore	National University of Singapore, Singapore City	**Perfect:** Analyses of DILI cases in health record databases revealed that the most commonly used causality assessment method was RUCAM, correctly quoted.
Tillmann et al., 2018	USA	East Carolina University, Greenville, NC	**Encouragement:** Among other points, the original RUCAM of 1993 is viewed as one of the most commonly used causality assessment tools that has become the standard for initial assessment of DILI causality. Authors are DILIN members, from various universities including South Carolina, Charleston, SC, or from the FDA NIDDK, Bethesda, NC.
Visentin et al., 2018	Switzerland	University Hospital Zurich, Zurich	**Promotion:** Perfect analyses of RUCAM of 1993 and the updated RUCAM of 2016 led to the statement that current diagnosis of DILI is based on the internationally harmonized RUCAM approach.
Cano-Paniagua et al., 2019	Colombia	University of Antioquia, Medellin	**Promotion:** RUCAM is favored and used for DILI cases providing highly probable and probable causality ranges in an excellent prospective study; the updated RUCAM was used and referenced, which allows discerning when confounding factors or concomitant drugs are present.
Becker et al., 2019	Brazil	Federal University of Health Science Porto Alegre	**Vague:** RUCAM was not used in their actual cohort but its use as the updated RUCAM of 2016 was recommended to establish causality in future cases.

DILI, drug-induced liver injury; MH cells, monocyte-derived hepatocyte-like cells; RUCAM, Roussel Uclaf Causality Assessment Method.

## RUCAM-DILI Case Quality (RDCQ)

The yield of highly qualified DILI reports of RUCAM-based cases could substantially be higher if some essentials would have been considered. In particular, the quality of present studies with RUCAM-based DILI cases is obviously variable regarding listed details of published cases (**Tables 4** and **5**). For instance, some reports had a retrospective study protocol instead of a preferred prospective one, and details occasionally were not reported such as the RUCAM version used, or results were incompletely provided. These shortcomings are, in principle, avoidable if studies are carefully planned prospectively. In an attempt to improve the quality of future DILI case publications, a list of essential items has been developed (**Table 8**). These items received a number of stars measuring the case or the study quality: the more stars the better the case or the study (**Table 8**). This tool may be called, in short, RDCQ (RUCAM-DILI Case Quality). Highly qualified reports may receive appreciation by 3 stars, whereas publications with an insufficient quality receive 2 or fewer stars. RDCQ provides a list of the most important elements required for planning studies on cases of suspected DILI and possible publication (**Table 8**). The first category comprises six essential elements, each awarded with 3 stars, while the second group consists of five elements, each of which may receive 2 stars, and the third category considers four elements, for each 1 star can be assigned. Accordingly, a quality ranking of intended DILI publications is achieved by summing up of the respective star numbers listed in front of each element of interest (**Table 8**). As a result, an excellent quality would be achieved with >28 stars, an acceptable one with 18–28 stars, and a disputable quality with <18 stars. Potential authors and editors are encouraged to use this qualifying scoring system.

**Table 8:** RUCAM-DILI Case Quality (RDCQ).

RUCAM-DILI Quality Stars	Obligatory elements required for presentation of DILI cases	Further details and comments for improving evaluation of DILI cases
***	Prospective clinical approach with prospective study protocol and the prospective use of the updated RUCAM version is mandatory. No question, emphasis is put on the prospective study and case management. Required is correct presentation of all data and references in the text.	Only prospective studies are of value, because they allow data collection at beginning and ensure complete data sets, which commonly provide high RUCAM causality gradings. Retrospective studies are of lower quality, conflicted by missing case data, allowing only low RUCAM causality gradings not sufficient to provide strong statements on results.
***	Mandatory is the use of RUCAM in its updated version only, with its mentioning in the text and listing among the references. Previous RUCAM versions are outdated and should not be used any more.	Since 2016, the updated RUCAM is the current version that should specifically be used and referenced (Danan and Teschke, 2016). Applying non-RUCAM method(s) as additional CAMs confound the results obtained by the updated RUCAM.
***	Presentation of the correct value of R (ratio) is essential to define the liver injury type using laboratory tests and no requiring liver biopsy results. The R value is needed for the selection and use of the correct RUCAM subscale, with description in the text and quotation in the reference list.	R is easily to be calculated through the multiple of the ULN of ALT divided by the multiple of the ULN of ALP. This allows differentiation of the hepatocellular injury (R > 5) from the cholestatic/mixed liver injury (R≤ 5). For both liver injury types specific RUCAM subscales are available and must be used for correct causality assessment, taking into account varying RUCAM scores.
***	Application of correct liver test thresholds for DILI is mandatory to exclude other liver diseases that are unrelated to drug therapy. Respective details and correct references belong in the text.	Thresholds for idiosyncratic DILI: ALT ≥5 × ULN and ALP ≥2 × ULN of hepatic origin. Values below the thresholds above signify liver adaptation or liver tolerance, those cases have to be excluded from analysis of the DILI case cohort.
***	Strict confinement to drugs known for causing idiosyncratic DILI is obligatory, thereby excluding other potentially hepatotoxic products; clarification in the text is essential.	If in the cohort of idiosyncratic DILI also cases of intrinsic DILI cases, HILI cases, or cases of liver injury by dietary supplements are included, this confounds the results obtained for the primary cohort.
***	Complete data, transparent and clear description of all data in the text with correct referencing is obligatory. Use of cases with probable and highly probable causality gradings for final results and discussion is essential.	Reports on DILI with incomplete essential data required for case understanding and possible re-evaluation are not useful for the scientific DILI and RUCAM community. Valuable are only well-documented DILI cases with a high causality grading based on evaluation by RUCAM.
**	Text presentation of final RUCAM scores and associated causality gradings, including cases with possible causality gradings and their case numbers.	This ensures correct information instead of only mentioning that DILI cases had been assessed for causality by RUCAM, a vague information not appreciated by the DILI and RUCAM experts.
**	Problematic are DILI cases with RUCAM-based causality gradings if possible. Discussions in the text should include tentative causes of this poor condition, associated with clear recommendations how to prevent this in future cases.	Inclusion of DILI cases with only a possible causality grading would confound the results obtained with DILI cases and their causality gradings of highly probable or probable. In addition, high numbers of cases with a possible causality grading are mostly found in cases with missing data.
**	Alternative causes found at the beginning of the study or during the further course should be listed in the text, with exact specification of the alternative diagnosis and case numbers.	Alternative causes heavily confound the description of DILI case characteristics. The required listing in the text also will show that details of the study cases have carefully been examined. RUCAM helps search for alternative causes.
**	Lists of narratives in small case series with ≤10 cases or single case reports are appreciated, better provided within the text instead of supplementary data, or in DILI databases.	Narratives are extremely valuable, because many other case details can be presented for which space within the text is limited. Clearly, narratives can easily be presented in DILI databases but not in large case series.
**	Presentation of the RUCAM scale with listing of all individual RUCAM elements and achieved scores, to be presented in small case series with ≤10 cases or single case reports.	This is an essential part of a good DILI case presentation that increases the quality of a DILI report by providing additional details and increasing transparency. It also allows for checking of completeness of available RUCAM elements in each case.
*	List of cases with results obtained at the occasion of an unintentional re-exposure and assumed positive test result should be presented in the text including LTs before and during re-exposure.	DILI cases with clear re-exposure test results are rarely reported in a correct way because positivity is mostly assumed in the absence of provided specific criteria as published with the updated RUCAM (Danan and Teschke, 2016).
*	List of cases with liver injury due to herbs and dietary supplements should be included in the text, if respective exclusion criteria have been neglected.	A separate list of non-DILI cases is mandatory; results of these cases must be presented separately. Inclusion in the DILI cohort would confound the DILI results as overall results were not those of real DILI.
*	As for most scientific publications, a summary of limitations of the report is mandatory and should be part of the text.	Important mandatory statement, initially often forgotten but later included upon request by a reviewer. Statement reflects critical view of own work.
*	Inclusion of a diagnostic flow chart in the text is not only informative but also stimulating. It improves the quality and readability of publications.	Such flow charts are appreciated by the readers facilitating a quick overview on details of the study. It makes a search of relevant results in the text of the publication unnecessary.

Quality assessment of DILI publications is achieved by summing up of the respective star numbers listed before each element of interest. Therefore, an excellent quality would be achieved with >28 stars, an acceptable one with 18–28 stars, and a disputable quality with <18 stars.

DILI, drug-induced liver injury; RDCQ, Roussel Ulaf Causality Assessment Method—Drug-Induced Liver Injury Case Quality, or in short RUCAM DILI Case Quality; RUCAM; Roussel Uclaf Causality Assessment Method.

## Alternative Causes and RUCAM

RUCAM plays also an investigative role to critically screen for potential alternative causes in cases of assumed DILI, as outlined in a recent analysis of a large case study published in 2018. Frequency and type of alternative causes were evaluated in cohorts of case series provided by 22 publications (**Table 9**) (Teschke and Danan, 2018c). Presented primarily as DILI in these publications, only part of the cases turned out to be real DILI cases after investigations including the use of a CAM such as RUCAM in search for verification or exclusion of nondrug causes (Teschke and Danan, 2018c). In all referenced publications, many cases with initially assumed DILI were reported that were not DILI.

**Table 9:** Alternative causes in initially assumed DILI cases.

Specific alternative causes	Cases (n)	Frequency (%)
Biliary diseases Autoimmune hepatitis Hepatitis B or C Tumor Ischemic hepatitis Hepatitis E Systemic sepsis Liver injury by other comedication Virus hepatitis Past liver transplantation Alcoholic liver Fatty liver Non-alcoholic steatohepatitis Hepatitis C Cardiac hepatopathy Thyroid hepatopathy Primary sclerosing cholangitis Primary biliary cholangitis Gilbert syndrome CMV Hepatitis EBV Hepatitis Hemochromatosis Wilson disease Paracetamol overdose Postictal state disease Osseous disease Lymphoma Preexisting liver cirrhosis Hepatitis B Benign recurrent intrahepatic cholestasis Rhabdomyolysis Polymyositis Chlamydial infection HIV infection	39 35 28 26 24 20 20 19 18 17 16 9 9 6 5 4 3 3 3 2 2 2 2 2 2 2 2 2 1 1 1 1 1 1	11.89 10.67 8.54 7.93 7.32 6.10 6.10 5.79 5.49 5.18 4.88 2.44 2.44 1.83 1.52 1.22 0.92 0.92 0.92 0.61 0.61 0.61 0.61 0.61 0.61 0.61 0.61 0.61 0.31 0.31 0.31 0.31 0.31 0.31
Total alternative cases	n = 328	100%

CMV, cytomegalovirus; DILI, drug-induced liver injury; EBV, Epstein–Barr virus.

### Frequency

The 22 publications reported on overall 13,336 cases. In 4,556/13,336 cases corresponding to 34.2%, alternative causes unrelated to DILI were presented; in other words, in more than one third of the cases, there was a potential alternative explanation for the hepatic disease, conditions not acceptable for the involved patients (Teschke and Danan, 2018c). The data on the 13,336 cases have been published by authors from different areas and countries, while reports were mostly from Asia, some came from the US, and less reports were published on cases from Europe. Overall, alternative diagnoses in the case series ranged from 4% up to 47%.

### Types of Alternative, Not Drug-Related Causes

Although most reports presented their alternative drug unrelated causes with specific diagnoses, many of the remaining publications provided only case numbers with alternative causes lacking diagnostic specifications. The 328 cases with specified non-drug causes are provided (**Table 9**) and had been submitted to further analysis (Teschke and Danan, 2018c).

#### Bile Tract and Intrahepatic Biliary Diseases

Biliary diseases were among the most alternative diagnoses, accounting for almost 12% (**Table 9**). These included, for instance, bile tract disorders causing biliary obstruction such as choledocholithiasis or infections clinically described as cholangitis. With these bile tract diseases, patients are at a high risk if the appropriate surgical intervention, endoscopic therapy, or treatment with antibiotics was withheld or delayed. Among other unrecognized hepato-biliary diseases were primary biliary cholangitis, a disease affecting the liver, and primary sclerosing cholangitis, with possible localization in the extrahepatic biliary tract system and intrahepatic localization (**Table 9**). Patients with these forms of hepato-biliary diseases commonly respond well upon initiation of specific drug therapies.

#### Liver Diseases

A variety of liver diseases unrelated to the use of drugs escaped the diagnostic efforts of the treating physicians (**Table 9**) (Teschke and Danan, 2018c). Among the not recognized alternatives diseases of the liver were mostly virus infections like hepatitis B, C, and E, while virus hepatitis by cytomegalovirus (CMV) and Epstein–Barr virus (EBV) were rarely described (**Table 9**). With hepatitis A virus (HAV) infection as another form of virus hepatitis, this diagnosis was not described as a missed cause in any of the published cases, but this is typical text book diagnosis nor requiring much expertise (Teschke and Danan, 2018c). Higher risks exist for patients suffering from ischemic hepatitis and cardiac hepatopathy, because these diseases would have better been treated under the care of cardiologists, as these liver diseases were the consequence of cardiopulmonary disorders and have therefore their origin outside the liver. Missing the diagnosis of an autoimmune hepatitis is another problem because for this liver disease, a specific corticosteroid therapy is strongly indicated provided the diagnosis has validly been established (**Table 9**). Hemochromatosis or Wilson’s disease is a typical genetic liver disease and was rarely described as missed diagnosis in the study cohort, unlike the more frequent non-alcoholic fatty liver disease (NAFLD), non-alcoholic steatohepatitis (NASH), and alcoholic liver disease (ALD), which all together amount to 10.4% of the alternative causes (**Table 9**). Clinical differentiation from DILI is usually not a problem on the basis of careful analysis, the clinical context, and if RUCAM is used for DILI verification or exclusion.

#### Other Drug Unrelated Causes as Alternative Diagnoses

Reports have been published on cases with serum bilirubin values above the normal range together with normal LTs, conditions suggestive of Gilbert syndrome, but in some instances, these laboratory alterations have erroneously been ascribed to DILI while the diagnosis of Gilbert syndrome was missed and therefore not published (**Table 9**) (Teschke and Danan, 2018c). Additionally, increased LTs were wrongly attributed to DILI in patients who experienced hypothyroidism, thyrotoxicosis, rhabdomyolysis, polymyositis, and postictal state (**Table 6**). Difficult to reconcile in other patients, enhanced ALP activities in the serum were wrongly attributed to DILI of the cholestatic type instead of underlying osseous diseases with fractures or metastases (**Table 9**). Again as a reminder for RUCAM users, in patients with increased serum ALP values, a non-hepatobiliary disease as cause has to be excluded using additional laboratory test as outlined above and previously (Danan and Teschke, 2016). DILI was also not correctly diagnosed in patients with malignant disorders like lymphoma that were responsible for almost 9% of the alternative, nondrug causes, or in patients with sepsis accounting for around 6% (Teschke and Danan, 2018c). It is clear from these data that in patients with increased LTs many alternative causes were insufficiently looked for. Although RUCAM helps exclude other causes that are defined as a special key element, it is recommended to work up an additional list of differential diagnosis, which has been published with the updated RUCAM (Danan and Teschke, 2016).

## Diagnostic Flow Chart

Diagnostic flow charts facilitate the quick catching of the presented data, for which respective references were provided earlier (Teschke and Danan, 2018c). The diagram helps the investigator at planning a prospective DILI study, and at its termination, it allows for a clear presentation of details obtained during the course of the study. This diagram should be submitted along with the manuscript to the journal as it enables the reader to gain a quick overview on the various steps during the course of the study. The suggested proposal should be viewed as a guidance allowing for modifications if necessary (**Figure 2**).

On top of the diagram, all cases of suspected idiosyncratic DILI have to be presented, with details of case numbers and criteria for exclusion and inclusion (**Figure 2**). Only cases with idiosyncratic DILI should be considered, excluding *a priori* all patients with intrinsic DILI and those with liver injury due to herbs or dietary supplements. Clearly, in analogy with conventional drugs as culprits of idiosyncratic DILI, herbs and dietary supplements can cause liver injury commonly viewed as HILI (herb-induced live injury). Characteristics of the two cohorts DILI and HILI differ substantially from each other and have to be evaluated separately in order to avoid results that are mixed up and thereby confounded. During the study, cases that do not meet the basic requirements for a good causality assessment should be excluded. Alternative causes and low causality gradings also should lead to case exclusion. At the end, a series of idiosyncratic DILI cases remains with uniform characteristics, which can easily be evaluated.

## Top Drugs Implicated in Causing DILI Assessed by RUCAM

Among the list of DILI case series (**Table 4**), there was also a study on 3,312 RUCAM-based DILI cases, assessed for drugs most commonly implicated in causing DILI (Teschke and Danan, 2018c). This study was triggered by previous publications describing poor quality of DILI case data, the inability to find enough DILI cases with established causality, and the associated problem of listing the most common drugs implicated in causing DILI (Björnsson, 2016a; Björnsson and Hoofnagle, 2016b). These problems started with expert analyses of DILI cases originating from the US LiverTox database, whereby part of the cases had been reassessed by retrospective use of the updated RUCAM (Björnsson, 2016a) or the original RUCAM (Björnsson and Hoofnagle, 2016b). Their expertise showed that many of the LiverTox cases of DILI could not be verified as real DILI, a difficult outcome for the LiverTox database, and also frustrating for the scientific DILI community. The results of these investigations suggest a more cautious interpretation of the DILI cases online available at the LiverTox database. Presently, there is no public statement whether and when this shortcoming will be addressed and what kind of causality assessment approaches will be undertaken to solve these problems. The use of the updated RUCAM might be a good idea.

Closely associated with these database issues was the problem to establish a top ranking of potentially hepatotoxic drugs. To solve this issue, an attempt was undertaken using 48 LiverTox database drugs with more than 50 case reports, for which a firm diagnosis of DILI by high-ranking hepatotoxic drugs has arbitrarily been assumed (Björnsson, 2016a). Using merely these numbers of case reports as the sole criterion for a drug ranking could result in data lacking accuracy. Perhaps a better approach was the use of DILI cases that had been evaluated for causality using RUCAM, available in the DILI databases of several registries and medical centers (Teschke, 2018d). These well-organized databases provided 3,312 drugs overall suitable for establishing a new top ranking that differed substantially from the previous LiverTox-based analysis (Teschke, 2018d). The new ranking of the 10 leading drugs involved in DILI is as follows, from top to bottom: Amoxicillin-clavulanate, Flucloxacillin, Atorvastatin, Disulfiram, Diclofenac, Simvastatin, Carbamazepine, Ibuprofen, Erythromycin, and anabolic steroids (**Table 10**).

**Table 10:** Ranking of drugs causing DILI with causality assessment cases by RUCAM.

Drug	RUCAM-based DILI cases (n)
1. Amoxicillin-clavulanate 2. Flucloxacilllin 3. Atorvastatin 4. Disulfiram 5. Diclofenac 6. Simvastatin 7. Carbamazepine 8. Ibuprofen 9. Erythromycin 10. Anabolic steroids 11. Phenytoin 12. Sulfamethoxazole/Trimethoprim 13. Isoniazid 14. Ticlopidine 15. Azathioprine/6-Mercaptopurine 16. Contraceptives 17. Flutamide 18. Halothane 19. Nimesulide 20. Valproate 21. Chlorpromazine 22. Nitrofurantoin 23. Methotrexate 24. Rifampicin 25. Sulfazalazine 26. Pyrazinamide 27. Gold salts 28. Sulindac 29. Amiodarone 30. Interferon beta 31. Propylthiouracil 32. Allopurinol 33. Hydralazine 34. Infliximab 35. Interferon alpha/Peginterferon 36. Ketaconazole 37. Busulfan 38. Dantrolene 39. Didanosine 40. Efavirenz 41. Floxuridine 42. Methyldopa 43. Minocycline 44. Telithromycin 45. Nevirapine 46. Quinidine 47. Sulfonamides 48. Thioguanine	333 130 50 48 46 41 38 37 27 26 22 21 19 19 17 17 17 15 13 13 11 11 8 7 7 6 5 5 4 3 2 1 1 1 1 1 0 0 0 0 0 0 0 0 0 0 0 0

Modified from a previous publication (Teschke, 2018d). Listed are the top-ranking 48 drugs causing DILI with verified causality using RUCAM.

In agreement with the results of older DILI studies, Amoxicillin-clavulanate is the leading drug combination in DILI (**Table 8**) (Teschke, 2018d). Liver injury is likely more related to the Clavulanate part than to Amoxicillin. Assessing the individual hepatotoxic risk of this drug combination, other details of drug use are essential such as daily dose and duration of treatment. In order to reach at a final conclusion on risk assessment and risk management, assessments in various countries with the drug specificities are needed, as well as a comparison with other potentially hepatotoxic antibiotics. Nevertheless, this example shows that such top rankings may have pragmatic consequences. Compiled from results included in various databases of many countries (**Table 10**), this new drug ranking provides only a global overview but does not replace considerations on specificities of individual countries with a different disease spectrum, including or excluding, for instance, tuberculosis, causing variabilities of drug use and associated DILI (Teschke, 2018d). A good example is Germany with a special ranking of drugs implicated in DILI, starting on top with Flupirtine, Clarithromycin, Fluoroquinolones, Estrogen + Diogenoest, Irbesartan, Terbinafine, and Metamizole. Conditions are different in China, where DILI is most frequently reported following therapy with antibiotics, antituberculosis drugs, antithyroid drugs, antineoplastic drugs, hypolipidemic drugs, antipyretic analgesics, antiepileptics, hypoglycemic drugs, antivirals, glucocorticoids, antithrombotics, and antihypertensive drugs. India is of special interest, where the top drugs are antituberculosis medications, more so than antiepileptic drugs, complementary and alternative medicine, antiretroviral drugs, and non-steroidal anti-inflammatory drugs. Experienced clinicians in a specific region usually know the most likely cases of liver injury caused by the drugs used in the same region. National regulatory agencies or specialized DILI registries usually can provide additional information on DILI specificities in a certain region or country.

Good data quality can be assumed for the currently listed drugs derived from DILI cases because most of these were provided by registry and clinical studies with a prospective study protocol using RUCAM early in the assessment approach (**Table 10**) (Teschke, 2018d). This is an important point to be reiterated because the prospective use of RUCAM facilitates early collection of complete data that allow for desired high-causality gradings of highly probable or probable. Apart from these advantages, prospective studies using RUCAM allow for homogeneity of study cohorts, in which real DILI cases are included not confounded by cases with alternative diagnosis that have nothing to do with drugs and related liver injury (Danan and Teschke, 2016; Teschke and Danan, 2018c).

## Biomarkers, DILI, and RUCAM

For confirming the diagnosis of idiosyncratic DILI in future suspected cases, a new and valid biomarker would be highly appreciated by the DILI community. For this purpose, there is an urgent need having case series at hands with DILI cases that have a valid diagnosis based on the use of a validated CAM (Teschke et al., 2017b), best achieved by using RUCAM (Danan and Teschke, 2016) and putting aside disputed subjective global introspection or opinion-based unstructured vague approaches lacking defined elements and respective scores (Teschke and Danan, 2018b).

In collaboration with IMI (Innovative Medicines Initiative) projects and more precisely the SAFE-T (Safer and Faster Evidence-based Translation) consortium, EMA (European Medicines Agency) presented a letter for support of DILI biomarker, addressing again various clinical and regulatory issues related to biomarkers in the setting of DILI (EMA, 2016). The focus was on the unavailability of clinical tests, sensitive and specific enough to validly establish the diagnosis of idiosyncratic DILI, and also to predict and monitor its clinical course. It was mentioned that current tools were limited and considered as a major hurdle in drug development if questions of liver injury are to be answered. Consensus exists among DILI experts that for clinical trials, specific DILI biomarkers have to be developed, with desired characteristics as outlined by EMA: 1) early or earlier detection of DILI as compared to current methods, 2) usability to predict DILI outcome and risk of severe DILI including acute liver failure, 3) monitoring of progression and regression of DILI, 4) assessment of liver adaptation as opposed to liver injury, and 5) searching for early intrinsic liver injury in clinical trials. It is of note that several heavily promoted biomarkers received critical comments in the past (Fontana, 2014), which is discussed in detail previously (Teschke et al., 2017b). A recent report on biomarkers focused on GLDH (Glutamate dehydrogenase) and provided an update of current knowledge in the DILI field (Church et al., 2019).

The EMA letter also called for biomarkers that could be used for early diagnosis of idiosyncratic DILI (EMA, 2016; Teschke et al., 2017b), with focus on CK-18 (Cytokeratin-18), microRNA-122 (microarray RNA-122), total HMGB-1 (High Mobility Group Box protein-1), GLDH, SDH (Sorbitol dehydrogenase), which is proposed as a marker for hepatocyte necrosis, and ccCK-18 (caspase-cleaved CytoKeratin-18), which is proposed as a marker for apoptosis (**Table 9**). Hyperacetylated HMGB-1 and MCSFR-1 (Macrophage colony-stimulating factor receptor-1) were proposed as markers for immune activation (**Table 9**) (EMA, 2016). Additional considerations focused on critically viewed M-30 (apoptosis), M-65 (apoptosis/necrosis), and microRNA-192 (unspecified liver damage) (Fontana, 2014). A recent report on proteomics analysis of monocyte-derived hepatocyte-like cells is of potential interest as it identified integrin beta 3 as a specific biomarker for DILI by diclofenac (Dragoi et al., 2018), but some basic questions are awaiting clarification as briefly mentioned (**Table 4**). However, a major problem emerged on 15 April 2019 that has only partially reached the DILI community, requiring new considerations of the above points on diagnostic biomarkers and partial retractions of previous claims in a variety of publications. Indeed, there is scientific misconduction of the lead investigator who characterized the marker hyperacetylated HMGB-1 (EMA, 2019). Further clarifications are needed for final conclusions, which is certainly outside the scope of the current article.

**Figure 3:**
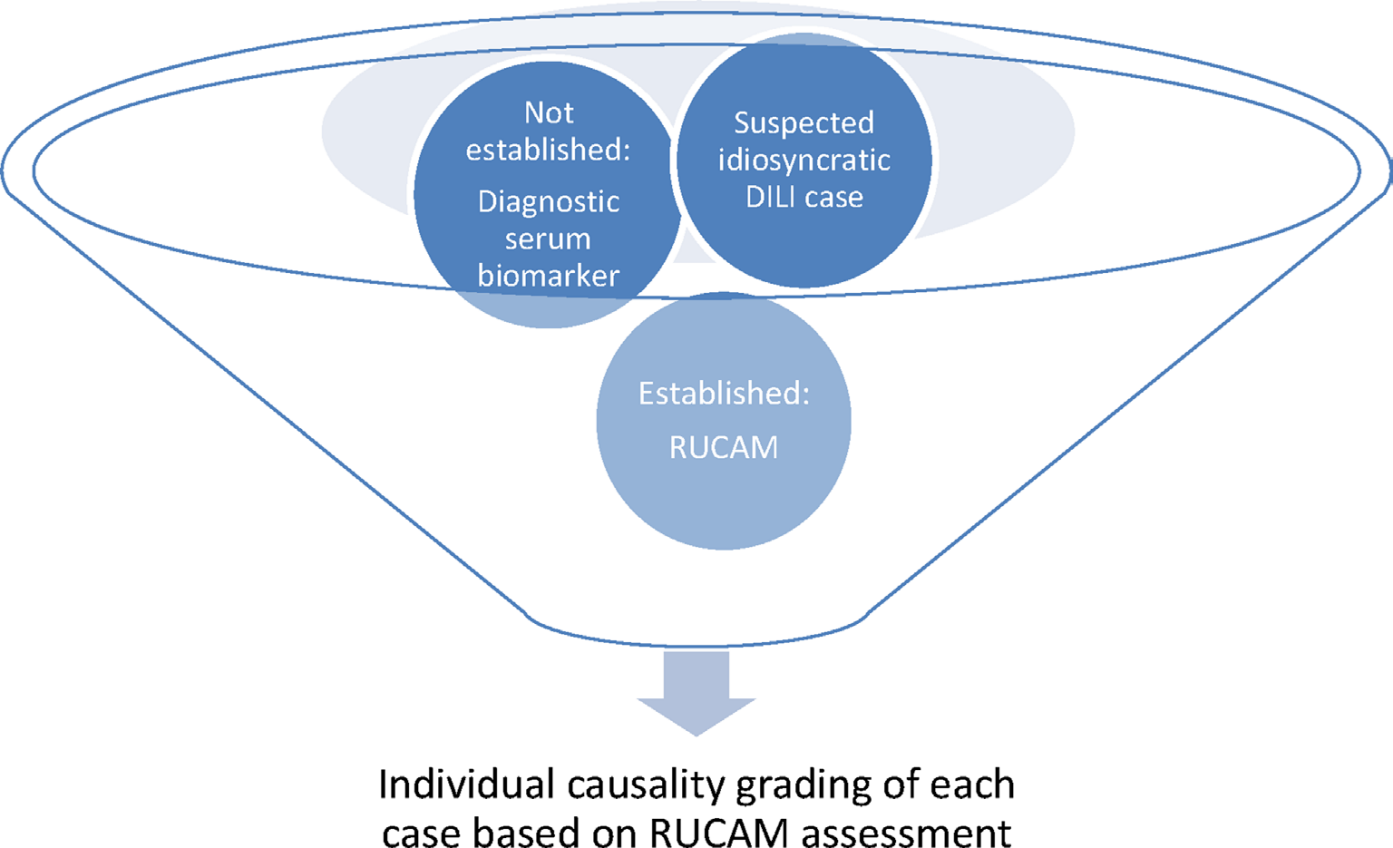
Valid causality assessment of idiosyncratic DILI using the established approach of RUCAM in the absence of a validated diagnostic serum biomarker. DILI, Drug induced liver injury; RUCAM, Roussel Uclaf Causality Assessment Method.

Considering the number of potential biomarkers and the variability of clinical and mechanistic targets (Teschke et al., 2017b; Church et al., 2019), it will be difficult to find at least one biomarker meeting all the essential requirements such as specificities for liver injury and among the around 1,000 drugs, which are potentially dangerous to the liver. As it presently stands, the situation remains unclear if, on the one hand, the suspected idiosyncratic DILI case is not validated and, on the other hand, the so-called established diagnostic serum marker is far from being validated. So the current solution of this dilemma is the use of RUCAM, which is the only element among the three items that is well established and enables an individual causality assessment of the suspected DILI case (**Figure 3**). Therefore, the medical problem is certainly not solved presently through a biomarker but *via* a diagnostic algorithm if RUCAM is used.

Occasionally, claims have been made that results obtained from genetic studies like those of HLA (human leucocyte antigen) genomes would represent diagnostic biomarkers of idiosyncratic DILI. This is certainly not correct because genetic data may represent risk factors but not diagnostic biomarkers. Prerequisite for both diagnostic biomarkers and risk factors is the need that is being derived from case series of RUCAM-based idiosyncratic DILI with high causality gradings like probable or highly probable.

## Conclusions

Idiosyncratic DILI is a fascinating but challenging disease if it comes to clearly establishing its diagnosis. This requires intuition, expertise, and the use of a robust CAM such as RUCAM available in the updated version published in 2016. Even non-supporters of RUCAM from the US reportedly acknowledge that RUCAM is the most widely used CAM for assessing causality in suspected DILI cases. Alone within a short period from 2014 until early 2019, DILI and RUCAM experts published more than 46,266 DILI cases that had been evaluated by using RUCAM. A substantial number of DILI experts including DILIN members initially not known for their RUCAM enthusiasm are now authors of publications on RUCAM-based DILI cases, thereby promoting the idea of the universal use of RUCAM. There is presently no other CAM with such success story based on a rigorous clinical approach of the suspected case, and no other CAM has evidently outperformed RUCAM. Problems are rarely reported in connection with the use of RUCAM in DILI, and if present, the problems were not found at the level of RUCAM itself but rather were related to the users. Indeed, the current analysis of the publications reporting on these 46,266 cases shows that RUCAM performs well provided that the RUCAM users do a good job.

Best RUCAM-based results on DILI cases can be achieved if users take care of the following points:

1. The study protocol should be prospective to ensure completeness of case data;
2. Complete data are necessary to achieve highly probable and probable causality gradings obtained using RUCAM, essential conditions for describing correct DILI features and risk factors, and increasing the likelihood to find biomarkers that are liver specific and could be drug specific;
3. Careful presentation and use of the R value, type of liver injury, and selection of the correct RUCAM subscale;
4. Uniformity of the study cohort consisting of patients with idiosyncratic DILI, excluding all patients with intrinsic DILI, HILI, liver injury by dietary supplements, and nondrug alternative causes;
5. Transparent data presentation, including final RUCAM causality gradings and scores;
6. Short narratives and list of scored RUCAM elements in single case reports and small case series ≤10 cases;
7. Presentation of a diagnostic flow chart diagram including alternative causes;
8. Quantitative evaluation of the study case quality using scores or stars of the RDCQ system;
9. Analysis and discussion of results derived from DILI cases with RUCAM causality gradings of highly probable or probable, eliminating cases with a possible or lower causality grading and all cases with alternative causes. In essence, RUCAM benefits from its wide use and performs well provided that the users adhere at established rules to prevent confounding variability.

## Author Contributions

The author confirms being the sole contributor of this work and has approved it for publication.

## Conflict of Interest Statement

The author declares that the research was conducted in the absence of any commercial or financial relationships that could be construed as a potential conflict of interest.
